# Repeated lidocaine exposure induces synaptic and cognitive impairment in aged mice by activating microglia and neurotoxic A1 astrocytes

**DOI:** 10.1016/j.isci.2025.112041

**Published:** 2025-02-17

**Authors:** Xiaohui Chen, Haiyang Wan, Yongxin Huang, Andi Chen, Xuyang Wu, Yanhua Guo, Jianjie Wei, Pinzhong Chen, Jiangdan Jiang, Xiaochun Zheng

**Affiliations:** 1 Department of Anesthesiology, Shengli Clinical Medical College of Fujian Medical University, Fujian Provincial Hospital, Fuzhou University Affiliated Provincial Hospital, Fuzhou, China; 2 Department of Anesthesiology, First Affiliated Hospital of Yangtze University, First People’s Hospital of Jingzhou, Jingzhou, China; 3 Fujian Emergency Medical Center, Fujian Provincial Key Laboratory of Critical Care Medicine, Fujian Provincial Co-Constructed Laboratory of “Belt and Road”, Fuzhou, China

**Keywords:** Pharmacology, Neuroscience, Immunology, Cell biology

## Abstract

In the perioperative setting, the administration of intravenous lidocaine is widespread. This study investigates the effects of varying frequencies of intravenous lidocaine on cognitive function in mice of differing ages. Young adult and aged mice received systemic lidocaine either once or three times. Our findings indicated that repeated exposure to systemic lidocaine in aged mice resulted in cognitive impairment, accompanied by neuronal apoptosis and synaptic loss in the hippocampus. Additionally, repeated lidocaine exposure activated microglia and neurotoxic A1 astrocytes in aged mice. Notably, the adverse effects were significantly diminished when aged mice were treated with dehydroxymethylepoxyquinomicin (DHMEQ), a specific NF-κB inhibitor. Furthermore, depleting microglia with PLX5622 effectively prevented the activation of A1 astrocytes and synaptic loss following lidocaine exposure. This study provides evidence linking age and exposure frequency to cognitive impairment due to systemic lidocaine administration, correlating with the activation of microglia and neurotoxic A1 astrocytes.

## Highlights

• The neurotoxicity of lidocaine is associated with age and the frequency of exposure
• Repeated lidocaine exposure leads to synaptic and cognitive impairment in aged mice
• Repeated lidocaine exposure activates microglia and neurotoxic A1 astrocytes
• The NF-κB pathway plays a crucial role in neurotoxicity induced by lidocaine

## Graphical abstract

**Figure:**
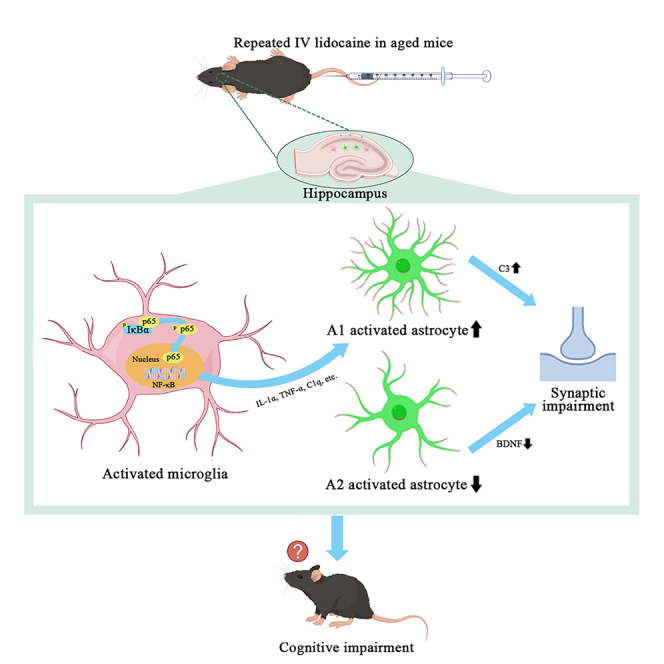

## Introduction

With the aging of the global population, it is estimated that up to 50% of elderly individuals will undergo one or more surgical procedures worldwide.^1^ Research has demonstrated that elderly patients with a range of conditions, such as intrahepatic and extrahepatic biliary stones, urolithiasis, bladder tumors, benign prostatic hyperplasia, and emergency trauma repair are susceptible to undergoing multiple surgeries within a limited time frame.^2^^,^^3^^,^^4^^,^^5^ Postoperative cognitive dysfunction (POCD) is a common neurological complication that often manifests after surgery and anesthesia, especially in the elderly population.^6^ POCD is characterized by either temporary or permanent cognitive decline, memory impairments, and difficulty adjusting to social situations, which potentially results in permanent disability and increased mortality of patients.^7^^,^^8^ The pathogenesis of POCD remains uncertain but is highly likely to be linked with the age of patients, the type of surgical operations, and the administration of anesthesia.^9^^,^^10^ Hence, when treating elderly patients, particular attention should be given to preventing anesthesia-induced cognitive dysfunction, considering the frequency of anesthesia and the amount and duration of perioperative anesthetics administered.

Lidocaine is a commonly used local anesthetic with various applications, such as surface anesthesia, infiltration anesthesia, nerve blocks, and intrathecal anesthesia.^11^ Recently, intravenous lidocaine has been widely used in the perioperative setting to alleviate postoperative pain and minimize opioid usage,^12^ speed up gastrointestinal function recovery,^13^ and even potentially improve tumor prognosis.^14^ However, it is important to recognize that systemic lidocaine has the potential to induce central nervous system (CNS) toxicity, which may lead to a variety of neurological complications, including oversedation, drowsiness, cognitive alterations, and, in severe cases, seizures, coma, and potentially death.^15^^,^^16^ Previous studies from various laboratories,^17^^,^^18^^,^^19^ including our own research,^20^ have shown that lidocaine can induce damage to neurons in the brain, spinal cord, and peripheral nerves in a time- and dose-dependent manner. Specifically, several animal studies suggest that the administration of lidocaine may induce neuroinflammation, which potentially involves the activation of immune cells.^21^^,^^22^ Moreover, the neurotoxic effects are significantly amplified with repeated exposure to anesthetics compared to a single exposure.^23^^,^^24^^,^^25^ Given the frequent necessity of multiple anesthesia and surgeries for elderly individuals to prevent recurring illnesses and uphold health, it is vital to investigate the effects of repeated lidocaine exposure on neurocognitive function at therapeutic levels, along with the underlying mechanisms.

Astrocytes, the most abundant cell type in the brain, play crucial roles in maintaining brain homeostasis and are involved in the neuroinflammatory response.^26^ In response to CNS injury and disease, astrocytes undergo changes in morphology and function, transforming into reactive cells that exhibit either a neurotoxic A1 phenotype or a neuroprotective A2 phenotype.^27^ Recent studies have shown that microglia in a classically activated state are capable of inducing the formation of reactive A1 astrocytes through the secretion of interleukin-1 alpha (IL-1α), tumor necrosis factor-alpha (TNF-α), and complement component 1q (C1q).^28^ Neurotoxic A1 astrocytes induced by microglial activation have been proposed as a significant contributor to age-related neurodegenerative disorders^29^ and the early onset of POCD.^30^ Therefore, further validation is required to investigate the effects of lidocaine exposure on microglial and astrocyte responses that are considered predominant neuropathological hallmarks of POCD.

Characterizing the dose- and age-related effects of intravenous lidocaine exposure is critical for creating safe clinical practices and providing informed preoperative counseling. Thus, the present study was performed to investigate the effects of different frequencies of lidocaine exposure (single vs. repeated) on neurocognitive function in different age groups (4 vs. 18 months old) of mice. Specifically, we investigated changes in cognitive function, synaptic plasticity, and the responses of microglia and astrocytes following lidocaine exposure. Additionally, we employed transcriptome sequencing and bioinformatics analysis to explore the molecular mechanisms underlying the neuropathological changes observed in the hippocampus. Our findings reveal that compared to a single exposure, repeated exposure of aged mice to lidocaine is highly neurotoxic. The differentially expressed genes enriched in biological processes were mainly involved in inflammatory responses and synaptic function. Notably, our results suggest the potential therapeutic benefits of dehydroxymethylepoxyquinomicin (DHMEQ), a specific inhibitor of NF-κB, in mitigating the adverse effects of repeated lidocaine exposure on cognitive function in aged mice. Furthermore, the depletion of microglia with PLX5622 successfully prevented the activation of neurotoxic A1 astrocytes and synaptic loss, emphasizing the promising role of targeted interventions in safeguarding neuronal integrity in response to lidocaine-induced neurotoxicity.

## Results

### Convulsive dose, plasma concentration and hemodynamic changes after the administration of lidocaine

**Figure 1:**
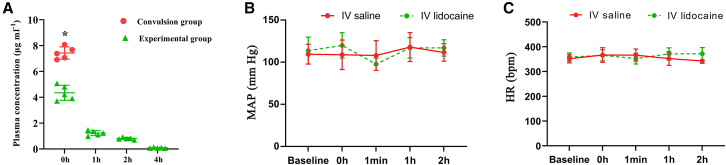
Plasma concentration and hemodynamic changes after the administration of lidocaine (A) Plasma concentrations of lidocaine following the administration of lidocaine (*n* = 5). (B) HR measured at baseline, immediately after administration, at 1 min, 1 h, and 2 h post-administration of lidocaine (*n* = 5). (C) MAP measured at baseline, immediately after administration, at 1 min, 1 h, and 2 h post-administration of lidocaine (*n* = 5).

In this study, we aimed to determine the median convulsive dose (CD50) of intravenous lidocaine in aged mice, which was found to be 22.07 mg kg^−1^. We also measured the plasma concentration of lidocaine to be 7.42 ± 0.47 μg mL^−1^ during the convulsive episodes. In the experimental group, the plasma concentration of lidocaine was assessed at 0 h, 1 h, 2 h, and 4 h after administering lidocaine using a bolus dose of 18.45 mg kg^−1^ followed by a continuous infusion of 12.30 mg kg^−1^ h^−1^ for a duration of 2 h. The measured plasma concentrations at these time points were 4.35 ± 0.58 μg mL^−1^, 1.24 ± 0.20 μg mL^−1^, 0.82 ± 0.08 μg mL^−1^, and 0.09 ± 0.03 μg mL^−1^, respectively (Figure 1A). The results clearly indicated that the experimental group had a noticeably lower plasma concentration of lidocaine, which is clinically relevant, compared to the concentration observed during convulsion at every time point. Furthermore, no convulsive behavior was observed, and the heart rate (HR) and mean arterial blood pressure (MAP) remained stable throughout continuous intravenous infusion in the experimental group (Figures 1A and 1B). These findings suggest that the administered dosage fell within the safe range. Accordingly, the dosage and administration route of lidocaine were selected as optimal for the subsequent studies.

### Repeated lidocaine exposure triggers cognitive impairment in aged mice but not adult mice

**Figure 2:**
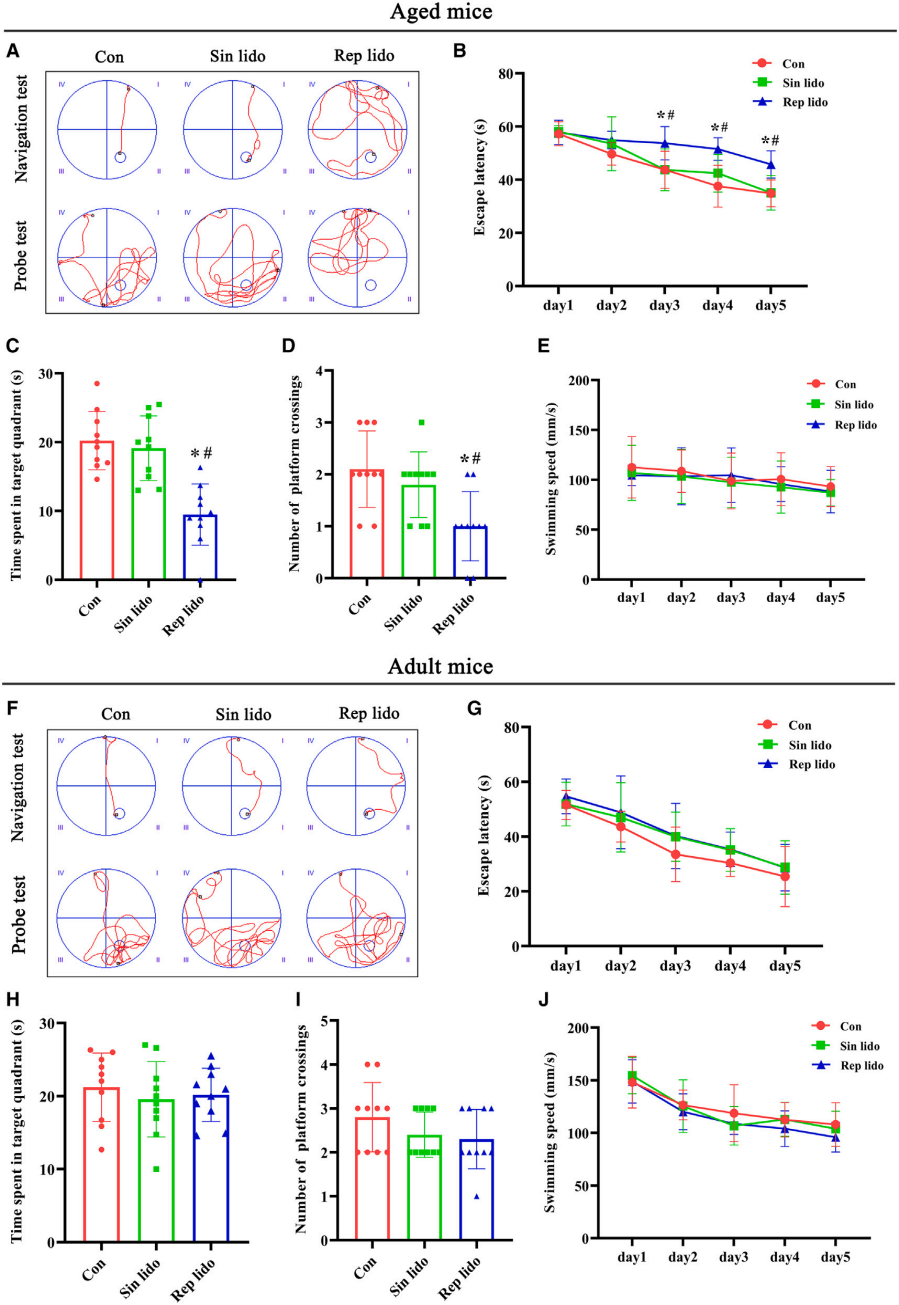
Repeated lidocaine exposure causes cognitive impairments in aged mice but not adult mice (A) Representative swim paths of aged mice on the fifth day of the place navigation and sixth day of the probe trial in the MWM test. (B) Escape latency of aged mice to find the hidden platform during the 5-day training trials. (C) Time spent in the target quadrant during the probe test. (D) Number of platform crossings of aged mice during the probe test. (E) Swim velocity of aged mice during the 5-day training trials. (F) Representative swim paths of adult mice on the fifth day of the place navigation and sixth day of the probe trial. (G) Escape latency of aged mice to find the hidden platform during the 5-day training trials. (H) Time spent in the target quadrant during the probe test. (I) Number of platform crossings of adult mice during the probe test. (J) Swim velocity of adult mice during the 5-days training trials. Con: control group; Sin lido: single lidocaine exposure group; Rep lido: repeated lidocaine exposure group. *n* = 10 per group. ∗*p* < 0.05 compared with the con group, ^#^*p* < 0.05 compared with the sin lido group. See also Figure S1.

We then confirmed the influence of different frequencies of intravenous lidocaine on cognitive function in mice of different ages. To do that, we established an animal model in which aged and adult mice were exposed to clinically relevant concentrations of lidocaine through single or repeated administration. The Morris water maze (MWM) test was utilized to evaluate cognitive function. Specifically, Figure 2A displays the typical swimming paths of the aged mice on the fifth day of the place navigation and sixth day of the probe trial. During the training session, all three groups of aged mice showed decreased escape latency over time, indicating an active learning process. However, aged mice that were repeatedly exposed to lidocaine spent more time finding the hidden platform during training days 2–5 compared to the control and single lidocaine groups (Figure 2B). During the probe trials, aged mice that were repeatedly exposed to lidocaine spent significantly less time in the target quadrant (Figure 2C) and had fewer platform crossings (Figure 2D) than the other two groups, indicating impaired memory performance. In addition, swim velocity was not significantly different among the groups (Figure 2E), suggesting that the differences in the MWM test results were induced by variations in cognitive function.

To further examine the role of age in lidocaine-induced cognitive impairment, adult mice received single or repeated doses of lidocaine. The MWM test revealed that in all three groups, the escape latency (Figure 2G), time spent in the target quadrant (Figure 2H), number of target crossings (Figure 2I), and swim velocity (Figure 2J) were not significantly different. These data demonstrate that cognitive impairment in aged mice can result from repeated exposure to lidocaine, rather than a single exposure. Sevoflurane, an inhalation anesthetic, is widely used in clinical anesthesia and has been linked to cognitive impairment, particularly in the elderly.^31^ Consistent with prior research, mice in the Rep sevo group exhibited prolonged search time for the hidden platform and reduced time spent in the target quadrant compared to Con group. Our MWM test results further indicated that repeated exposure to lidocaine somewhat worsened the working memory impairment induced by sevoflurane in aged mice, as evidenced by the Rep Sevo+Lido group spending less time in the target quadrant and making fewer platform crossings during the probe trials compared to the Rep sevo group (Figure S1).

### RNA-seq analysis revealed that DEGs in the hippocampus were enriched in inflammatory responses and synaptic signaling after repeated lidocaine exposure in aged mice

**Figure 3:**
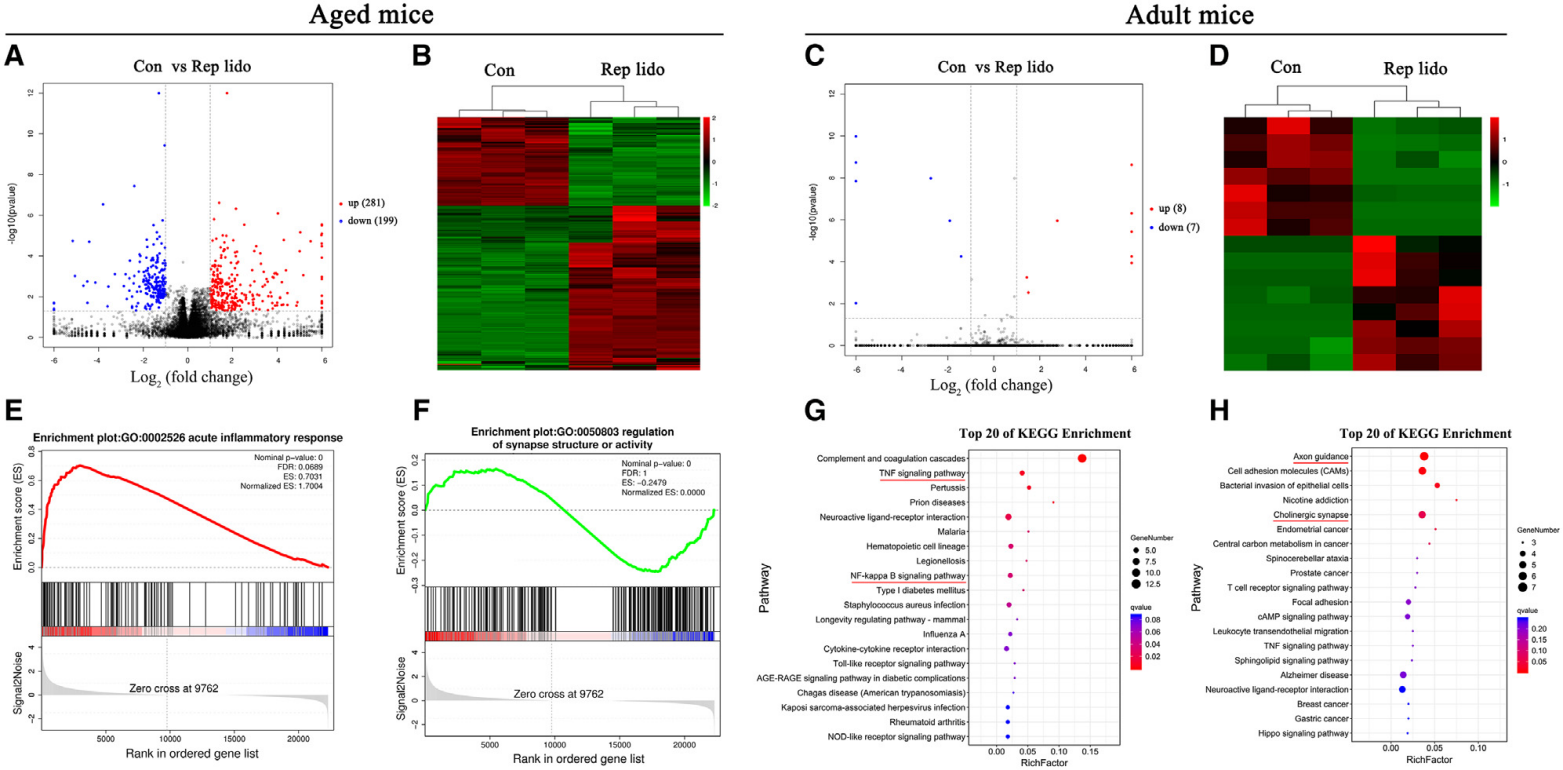
RNA-seq analysis indicated that DEGs in the hippocampus were enriched in inflammatory responses and synaptic signaling after repeated exposure to lidocaine (A) Volcano map of DEGs in aged mice. Red dots are upregulated genes, and blue dots are downregulated genes. (B) Heatmaps show the expression patterns of DEGs in aged mice. (C) Volcano map of DEGs in adult mice. (D) Heatmaps show the expression patterns of DEGs in adult mice. (E and F) Representative pathways enriched in the identified genes as determined by GSEA in the hippocampus of aged mice. The x axis represents the distribution of genes, from the most strongly upregulated to the most strongly downregulated. (G and H) KEGG pathway enrichment dot plot of the significantly upregulated and downregulated genes in the hippocampus of aged mice. Con: control group; Rep lido: repeated lidocaine exposure group. *n* = 3 per group.

Next, we performed RNA-seq analysis to identify genes and molecular pathways underlying lidocaine-induced cognitive impairment. In the hippocampus of aged mice that were administered lidocaine repeatedly, we identified a total of 480 genes with differential expression (DEGs). Specifically, 281 DEGs (59%) were found to be significantly upregulated, while 199 DEGs (41%) were significantly downregulated (Figures 3A and 3B). In contrast, adult mice repeatedly exposed to lidocaine did not show a significant difference in overall gene expression compared to the control group (Figures 3C and 3D).

After conducting the GSEA-GO analysis of the top and bottom NES rankings (top 20), it was noted that aged mice exposed to lidocaine repeatedly showed a notable increase in the acute inflammatory response (Figure 3E) and a considerable decrease in synaptic structure and activity (Figure 3F) in the hippocampus when compared to the control group. Furthermore, the significantly upregulated DEGs in the KEGG pathway analysis were found to be enriched in functional annotation related to tumor necrosis factor (TNF) and nuclear factor kappa B (NF-κB) signaling pathways (Figure 3G). Additionally, KEGG pathway analysis revealed that a majority of the downregulated DEGs were enriched in axon guidance and cholinergic synapses in the aged mice hippocampus 24 h after repeated exposure to lidocaine (Figure 3H). These data suggested that aged mice repeatedly exposed to lidocaine exhibited cognitive impairment, which was highly attributed to neuroinflammation and synaptic dysfunction in the hippocampus.

### Repeated lidocaine exposure induces microglial activation and upregulates the NF-κB pathway in the hippocampus of aged mice

**Figure 4:**
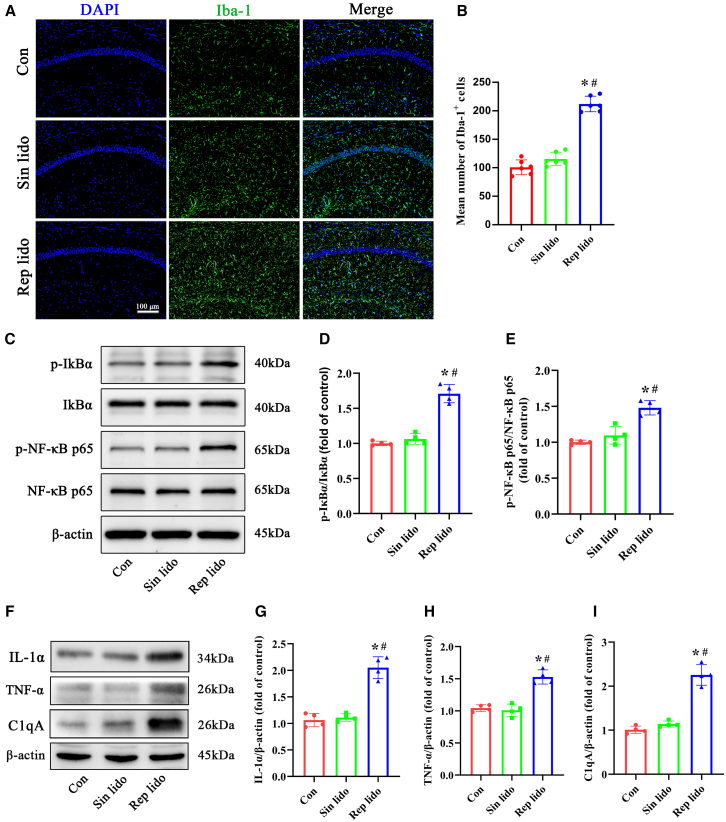
Repeated lidocaine exposure induces microglial activation, upregulates the NF-κB pathway, and increases proinflammatory cytokines in the hippocampus of aged mice (A and B) Representative pictures of immunofluorescence staining of microglia (labeled by Iba-1 in green) and cell nuclei (labeled by DAPI in blue) in the hippocampal CA1 region. The statistical chart presents the number of microglia per field for each group (*n* = 6, scale bar: 100 μm). (C–E) Representative western blot of *p*-IκBα, IκBα, p-NF-κB p65, and NF-κB p65 proteins in the hippocampus. Densitometric analyses of the immunoblots were performed, and the results are expressed as percentages relative to the control group (*n* = 4). (F–I) Representative western blot of proinflammatory cytokine IL-1α, TNF-α and C1qA proteins in the hippocampus. Densitometric analyses of the immunoblots were performed, and the results are expressed as percentages relative to the control group (*n* = 4). Con: control group; Sin lido: single lidocaine exposure; Rep lido: repeated lidocaine exposure. ∗*p* < 0.05 compared with the con group, ^#^*p* < 0.05 compared with the sin lido group. Data were presented as the mean ± SD.

We utilized Iba-1 immunofluorescence to track microglial activation. In Figure 4A, microglial cells in the hippocampus of control and single lidocaine-treated mice displayed smaller cell bodies with fewer and scattered processes. Conversely, microglia exhibited a reactive morphology in the hippocampus of mice repeatedly treated with lidocaine, characterized by enlarged cell bodies and thicker processes. Moreover, there was a noticeable increase in the number of Iba-1^+^ microglia in the hippocampus of aged mice following repeated lidocaine exposure compared to the control and single lidocaine groups. Western blot analysis further revealed an elevation in the levels of *p*-IκBα and p-NF-κB p65 in the hippocampus of aged mice after repeated lidocaine exposure, suggestive of NF-κB signaling activation (Figures 4C–4E). Additionally, the expression levels of the microglia-derived proinflammatory cytokines IL-1α, TNF-α, and C1qA were significantly higher in the repeated lidocaine group compared to both the control and single lidocaine groups (Figures 4F–4I).

### Repeated lidocaine exposure promotes the conversion of astrocytes to the A1 phenotype in the hippocampus of aged mice

**Figure 5:**
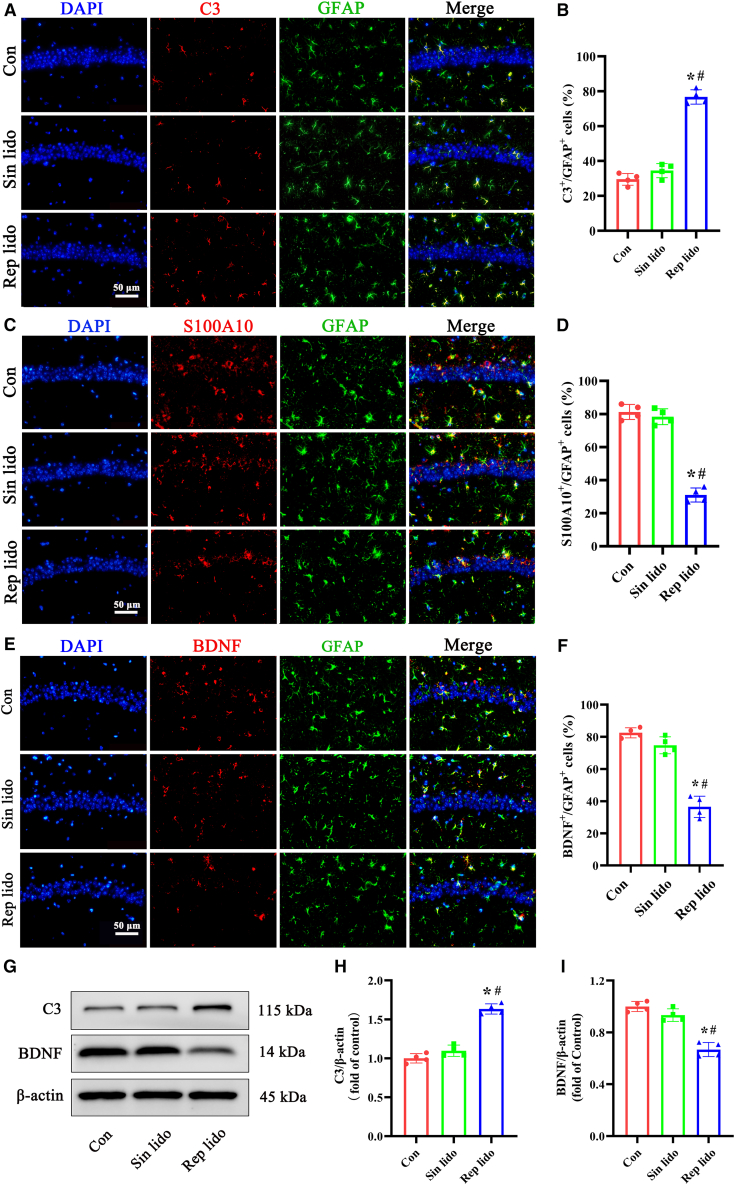
Repeated lidocaine exposure promotes the transformation of astrocytes from the A2 phenotype to the A1 phenotype in the hippocampus of aged mice (A and B) Representative pictures of immunofluorescence staining of astrocytes (labeled by GFAP in green), neurotoxic markers (labeled by C3 in red) and cell nuclei (labeled by DAPI in blue) in the hippocampal CA1 region. The statistical chart presents the number of C3/GFAP-positive cells per field for each group (*n* = 4, scale bar: 50 μm). (C and D) Representative pictures of immunofluorescence staining of astrocytes (labeled by GFAP in green), a neuroprotective marker (labeled by S100A10 in red) and the cell nucleus (labeled by DAPI in blue) in the hippocampal CA1 region. The statistical chart presents the number of S100A100/GFAP-positive cells per field for each group (*n* = 4, scale bar: 50 μm). (E and F) Representative pictures of immunofluorescence staining of astrocytes (labeled by GFAP in green), BDNF (red) and cell nuclei (labeled by DAPI in blue) in the hippocampal CA1 region. The statistical chart presents the number of BDNF/GFAP-positive cells per field for each group (*n* = 4, scale bar: 50 μm). (G–I) Representative western blot of C3 and BDNF proteins in the hippocampus. Densitometric analyses of the immunoblots were performed, and the results are expressed as percentages relative to the control group (*n* = 4). Con: control group; Sin lido: single lidocaine exposure; Rep lido: repeated lidocaine exposure. ∗*p* < 0.05 compared with the con group, ^#^*p* < 0.05 compared with the sin lido group. Data were presented as the mean ± SD. See also Figure S2.

We examined the presence of A1 astrocytes, identified by glial fibrillary acidic protein (GFAP) and complement component 3 (C3), as well as A2 astrocytes, marked by GFAP and S100A10, in the hippocampus of aged mice following lidocaine exposure. Immunofluorescence analysis revealed a significant increase in the number of C3/GFAP-positive astrocytes in the hippocampus after repeated lidocaine exposure compared to the control and single lidocaine groups (Figures 5A and 5B). Conversely, the number of S100A10/GFAP-positive astrocytes in the repeated lidocaine-treated group was significantly lower than that in the control and single lidocaine-treated groups (Figures 5C and 5D). Furthermore, RT‒qPCR results showed a significant increase in the levels of A1-specific genes (C3, H2-D1, Serping1, Amigo2) and a decrease in the levels of A2-specific genes (S100A10, CD14, Emp1, Stat3) in the hippocampus of aged mice after repeated lidocaine exposure (Figure S2).

Our immunofluorescence analysis demonstrated the colocalization of brain-derived neurotrophic factor (BDNF) and GFAP in astrocytes within the hippocampus. As depicted in Figures 5E and 5F, there was a notable decrease in BDNF/GFAP-positive astrocytes in the hippocampus following repeated lidocaine exposure compared to the control and single lidocaine groups. Additionally, western blot analysis revealed upregulation of C3 and downregulation of BDNF in the hippocampus of aged mice following repeated lidocaine exposure (Figures 5G–5I). Taken together, the results indicate that repeated exposure to lidocaine promotes the transformation of astrocytes from the neuroprotective A2 phenotype to the neurotoxic A1 phenotype, whereas a single exposure does not elicit the same effect.

### Repeated lidocaine exposure induced synaptic impairment and neuronal apoptosis in the hippocampus of aged mice

**Figure 6:**
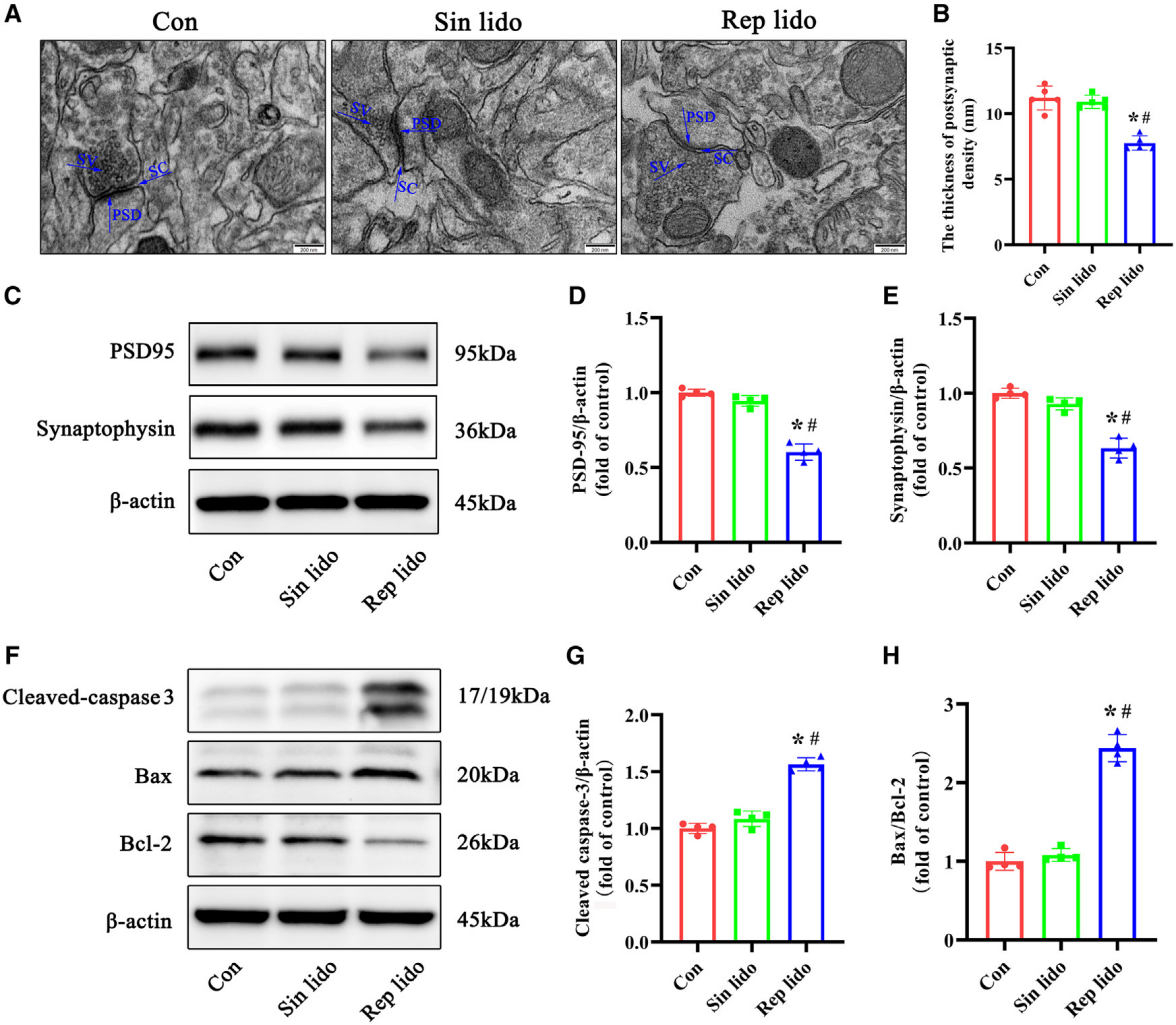
Repeated lidocaine exposure induced synaptic impairment and neuronal apoptosis in the hippocampus of aged mice (A and B) Representative photomicrograph (37000x) demonstrating variations in PSD thickness in the hippocampus. The statistical chart displays the PSD thickness from at least 20 sections for each group (*n* = 5, scale bar: 200 nm). (C–E) Representative western blot of PSD95 and synaptophysin in the hippocampus. Densitometric analyses of the immunoblots were performed, and the results are expressed as percentages relative to the control group (*n* = 4). (F–H) Representative western blot of Bax, Bcl-2, and cleaved caspase-3 in the hippocampus. Densitometric analyses of the immunoblots were performed, and the results are expressed as percentages relative to the control group (*n* = 4). Con: control group; Sin lido: single lidocaine exposure; Rep lido: repeated lidocaine exposure. ∗*p* < 0.05 compared with the con group, ^#^*p* < 0.05 compared with the sin lido group. Data were presented as the mean ± SD.

We examined synaptic morphological changes and the expression of synaptic markers (PSD95 and synaptophysin) using TEM and western blot analysis, respectively. Figures 6A and 6B illustrated a significant reduction in the thickness of PSD in the hippocampal CA1 area of aged mice repeatedly exposed to lidocaine compared to the control and single lidocaine-exposed mice. Furthermore, we observed a downregulation of PSD95 and synaptophysin at the protein level in the hippocampus of aged mice following repeated lidocaine exposure, as depicted in Figures 6C–6E. We also investigated the levels of cleaved caspase-3 and Bax/Bcl-2 ratio, as they are known to play a crucial role in the process of cell apoptosis. Results from western blot analysis confirmed a significant increase in the expression of the apoptosis-related proteins cleaved caspase-3 and Bax following repeated lidocaine exposure, accompanied by a decrease in the expression of Bcl-2 compared to the control and single lidocaine groups (Figures 6F–6H).

### Pharmacological depletion of microglia attenuated A1 astrocyte activation and synaptic impairment induced by lidocaine in the hippocampus of aged mice

**Figure 7:**
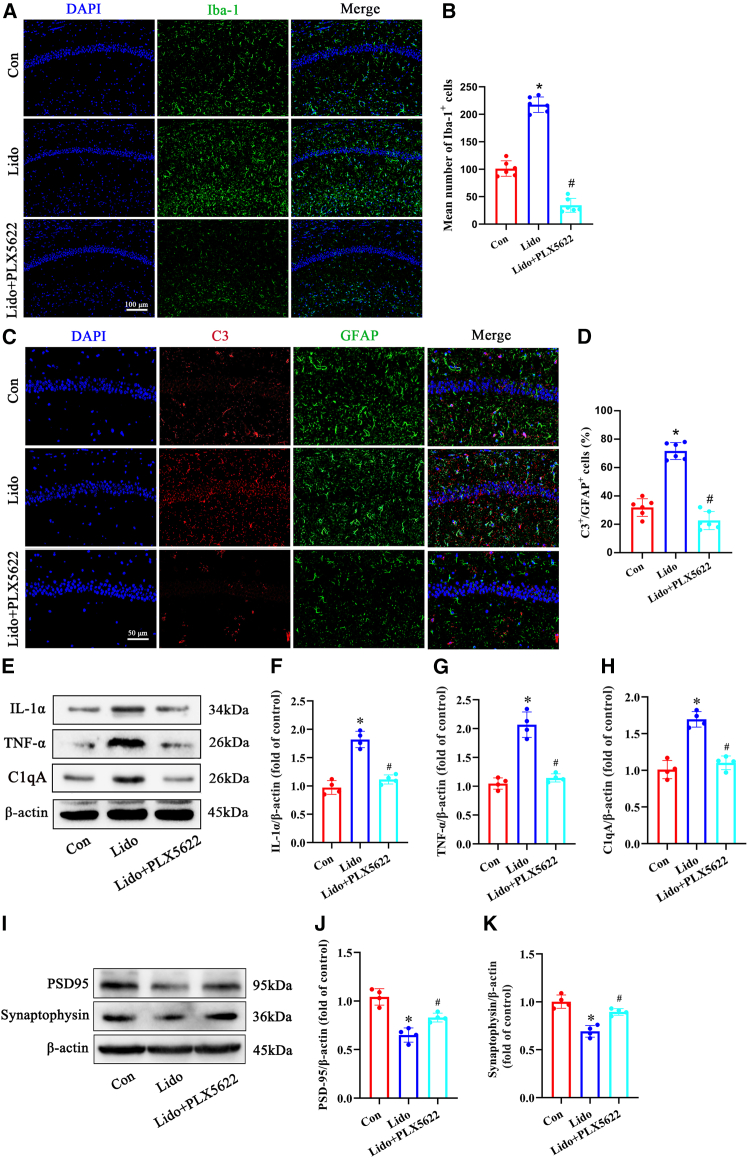
Microglial depletion by PLX5622 treatment attenuated A1 astrocyte activation and synaptic impairment induced by repeated lidocaine exposure in the hippocampus of aged mice (A and B) Representative pictures of immunofluorescence staining of microglia (labeled by Iba-1 in green) and cell nuclei (labeled by DAPI in blue) in the hippocampal CA1 region. The statistical chart presents the number of microglia per field for each group (*n* = 6, scale bar: 100 μm). (C and D) Representative pictures of immunofluorescence staining of astrocytes (labeled by GFAP in green), neurotoxic markers (labeled by C3 in red) and cell nuclei (labeled by DAPI in blue) in the hippocampal CA1 region. The statistical chart presents the number of C3/GFAP-positive cells per field for each group (*n* = 6, scale bar: 50 μm). (E–H) Representative western blot of proinflammatory cytokine IL-1α, TNF-α, and C1qA proteins in the hippocampus. Densitometric analyses of the immunoblots were performed, and the results are expressed as percentages relative to the control group (*n* = 4). (I–K) Representative western blot of PSD95 and synaptophysin in the hippocampus. Densitometric analyses of the immunoblots were performed, and the results are expressed as percentages relative to the control group (*n* = 4). Con: control group; Lido: group that received repeated lidocaine exposure. ∗*p* < 0.05 compared with the con group, ^#^*p* < 0.05 compared with the lido group. Data were presented as the mean ± SD.

To examine the potential impact of microglial activation on astroglial response and synaptic function in aged mice following repeated lidocaine exposure, we utilized PLX5622 to deplete microglia in the mice’s brains. As anticipated, mice in the PLX5622 treatment group exhibited a marked reduction in the microglial population compared to mice on a control diet following lidocaine exposure (Figures 7A and 7B). Furthermore, the levels of inflammatory cytokines, including IL-1α, TNF-α, and C1q, released by microglia were diminished in the hippocampus of aged mice after PLX5622 treatment (Figures 7E–7H). Subsequently, the astroglial response and synaptic function were assessed in aged mice treated with lidocaine, with or without PLX5622. Following repeated lidocaine exposure, there was a noticeable increase in the population of A1 astrocytes and a corresponding loss of synapses in the hippocampus. Interestingly, depletion of microglia significantly ameliorated the activation of A1 astrocytes induced by lidocaine (Figures 7C and 7D) and alleviated synaptic impairment in aged mice (Figures 7I–7K).

### Inhibition of the NF-κB pathway prevented microglial activation and A1 astrocyte polarization induced by lidocaine in the hippocampus of aged mice

**Figure 8:**
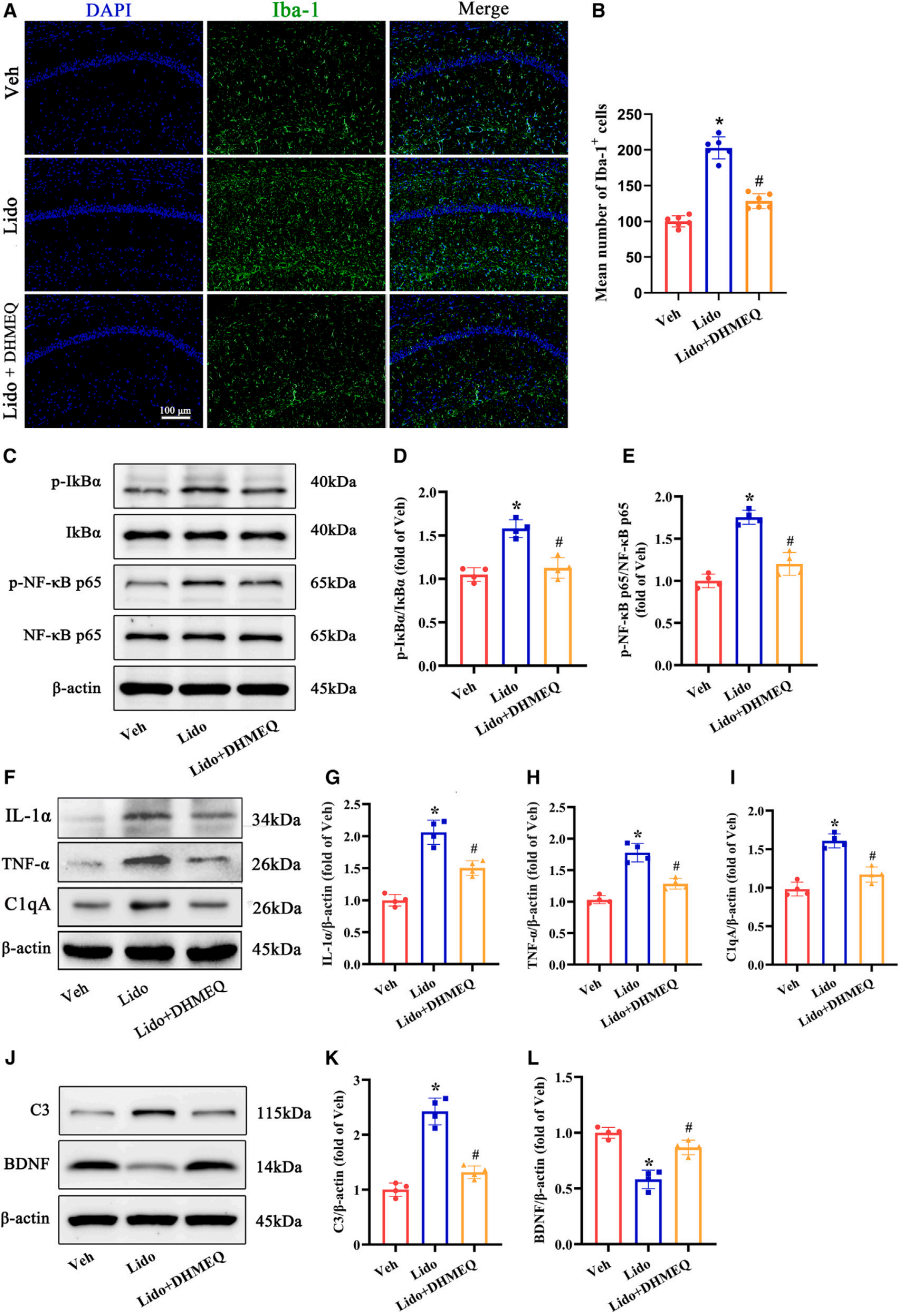
DHMEQ treatment prevented microglial activation and A1 astrocyte polarization induced by repeated lidocaine exposure in the hippocampus of aged mice (A and B) Representative pictures of immunofluorescence staining of microglia (labeled by Iba-1 in green) and cell nuclei (labeled by DAPI in blue) in the hippocampal CA1 region. The statistical chart presents the number of microglia per field for each group (*n* = 6, scale bar: 100 μm). (C–E) Representative western blot of p-IκBα, IκBα, p-NF-κB p65, and NF-κB p65 proteins in the hippocampus. Densitometric analyses of the immunoblots were performed, and the results are expressed as percentages relative to the control group (*n* = 4). (F–I) Representative western blot of proinflammatory cytokine IL-1α, TNF-α and C1qA proteins in the hippocampus. Densitometric analyses of the immunoblots were performed, and the results are expressed as percentages relative to the control group (*n* = 4). (J–L) Representative western blot of C3 and BDNF proteins in the hippocampus. Densitometric analyses of the immunoblots were performed, and the results are expressed as percentages relative to the control group (*n* = 4). Veh: vehicle group; lido: group that received repeated lidocaine exposure. ∗*p* < 0.05 compared with the veh group, ^#^*p* < 0.05 compared with lido group. Data were presented as the mean ± SD. See also Figure S3.

To investigate the involvement of NF-κB signaling in mediating microglial activation and the generation of A1 neurotoxic astrocytes after exposure to lidocaine, we employed the specific NF-κB inhibitor DHMEQ. Consistent with our prior results in Figure 4A, upon repeated lidocaine injections in aged mice, there was a significant increase in the number of IBA1^+^ microglia in the hippocampus, exhibiting active cell morphology characterized by enlarged cell bodies and thicker processes. Nevertheless, administration of the DHMEQ notably reduced microglial activation, resulting in fewer IBA1^+^ microglia with a more ramified morphology (Figures 8A and 8B). In addition, DHMEQ treatment decreased microglia-derived proinflammatory cytokines after repeated exposure to lidocaine, as indicated by a significant reduction in the expression levels of *p*-IκBα and p-NF-κB p65 (Figures 8C–8E), as well as a decrease in the expression levels of IL-1α, TNF-α, and C1qA (Figures 8F–8I). Moreover, when DHMEQ was administered to aged mice repeatedly exposed to lidocaine, the expression level of C3 significantly decreased to baseline, while the expression level of BDNF was remarkably rescued (Figures 8J–8L). As expected, the RT‒qPCR results revealed that DHMEQ treatment significantly decreased the levels of astrocyte A1-specific genes (C3, H2-D1, Serping1, Amigo2) in the hippocampus of aged mice following repeated lidocaine exposure while increasing the levels of A2-specific genes (S100A10, CD14, Emp1, Stat3) (Figure S3). These results support the idea that inhibition of the NF-κB signaling pathway is a plausible approach to mitigate neuroinflammation induced by lidocaine and prevent the neurotoxic polarization of astrocytes, thereby maintaining astrocytes in a neuroprotective phenotype.

### Inhibition of the NF-κB pathway attenuated synaptic loss and neuronal apoptosis and improved cognitive impairment induced by lidocaine in the hippocampus of aged mice

**Figure 9:**
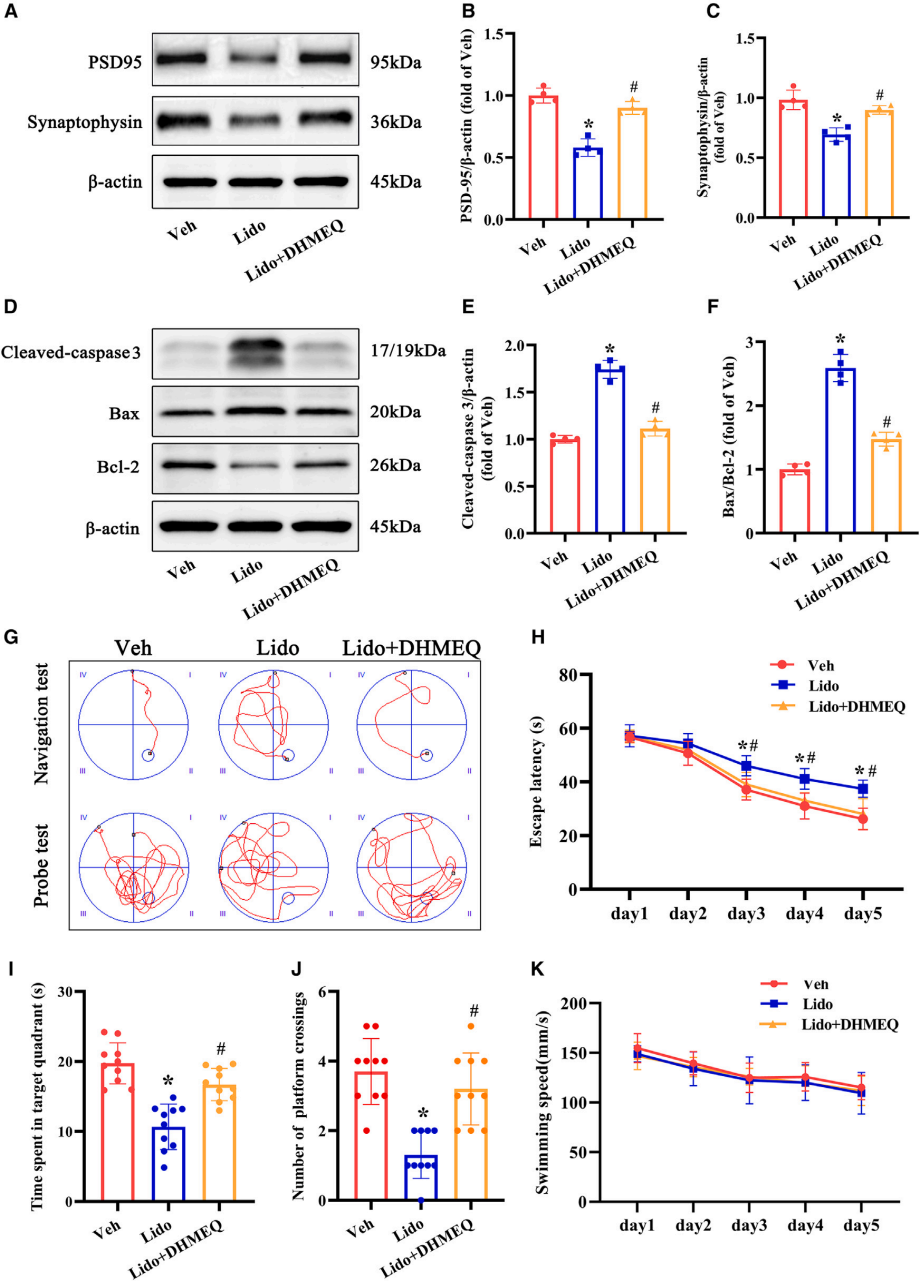
DHMEQ treatment alleviated synaptic loss and neuronal apoptosis and improved cognitive impairment triggered by repeated lidocaine exposure in the hippocampus of aged mice (A–C) Representative western blot of PSD95 and synaptophysin in the hippocampus. Densitometric analyses of the immunoblots were performed, and the results are expressed as percentages relative to the control group (*n* = 4). (D–F) Representative western blot of Bax, Bcl-2, and cleaved caspase-3 in the hippocampus. Densitometric analyses of the immunoblots were performed, and the results are expressed as percentages relative to the control group (*n* = 4). (G) Representative swim paths of aged mice on the fifth day of the place navigation and sixth day of the probe trial (*n* = 10). (H) Escape latency of aged mice to find the hidden platform during the 5-day training trials (*n* = 10). (I) Time spent in the target quadrant during the probe test (*n* = 10). (J) Number of platform crossings of aged mice during the probe test (*n* = 10). (K) Swim velocity of aged mice during the 5-days training trials (*n* = 10). Veh: vehicle group; lido: group that received repeated lidocaine exposure. ∗*p* < 0.05 compared with the veh group, ^#^*p* < 0.05 compared with the repeated lidocaine dose-treated group. Data were presented as the mean ± SD.

We next investigated whether the NF-κB signaling pathway is implicated in synaptic and cognitive impairment in aged mice subjected to repeated lidocaine exposure. DHMEQ treatment was found to reverse the significant reduction in PSD-95 and synaptophysin levels in the hippocampus of aged mice following lidocaine exposure, as shown by western blot analysis (Figures 9A–9C). Furthermore, repeated lidocaine exposure resulted in an increase in levels of cleaved caspase-3 and the Bax/Bcl-2 ratio, which were effectively mitigated by DHMEQ treatment in the hippocampus (Figures 9D–9F). Additionally, results from the MWM test demonstrated that DHMEQ treatment reduced the escape latency time (Figures 9G and 9H), significantly increased the time spent in the target quadrant (Figure 9I), and augmented the number of target crossings (Figure 9J) compared to the repeated lidocaine group. Swim velocity was not significantly different among the groups (Figure 9K). Taken together, these findings suggest that repeated exposure to lidocaine can lead to synaptic and cognitive impairments, primarily attributed to the activation of the NF-κB pathway.

## Discussion

Local anesthetic systemic toxicity (LAST) is a potentially life-threatening complication that should be carefully considered whenever local anesthesia is employed.^32^ Lidocaine remains the predominant local anesthetic, with a growing trend of intravenous use in the perioperative period. Previous animal and observational studies have indicated temporary cognitive impairment during convulsive episodes induced by the administration of local anesthetics.^33^^,^^34^ The fact that lidocaine affects the CNS is not surprising, considering its ability to easily cross the blood‒brain barrier. Nevertheless, the molecular mechanisms underlying the neurotoxic effects of systemic lidocaine in CNS are poorly understood. A previous study employing the 2-[14C] deoxyglucose technique to investigate lidocaine distribution in the rodent brain found that lidocaine concentration is uneven, with notable accumulation in the hippocampus—an area recognized for its role in learning, memory, and cognitive function.^35^ Even a single application of a subconvulsant dose of lidocaine still resulted in damage to the hippocampus and amygdala neurons in adult rats, confirming that lidocaine can potentially cause CNS injury even at “relatively safe” doses.^17^ Given the hippocampus’s notable sensitivity to systemic lidocaine,^17^^,^^35^ we chose to focus our study specifically on this brain region. We hypothesized that the effect of intravenous lidocaine on neurocognitive function could be influenced by the frequency of administration and the age of the patient. Accordingly, the present study aimed to evaluate the effects of different frequencies of intravenous lidocaine administered at a clinically relevant concentration on neurocognitive function across diverse age groups.

In this study, the choice of dosage and administration regimen of lidocaine was based on the dose-conversion correlation between humans and mice (humans: mice = 1: 12.3).^36^ The objective was to investigate the effectiveness and safety of administering lidocaine at a clinically relevant concentration using an animal model.^37^ Throughout the experiment, we carefully monitored the mice for any signs of adverse effects, such as convulsive behavior and cardiac toxicity. No significant signs of convulsions or hemodynamic changes were observed in any of the mice, even in mice with the highest lidocaine concentration, which remained below the previously reported toxic concentration following lidocaine administration.^38^ Our study demonstrated that repeated exposure to lidocaine, even at a clinically relevant concentration, induces cognitive impairment specifically in aged mice. Additionally, this impairment is correlated with neuronal apoptosis and synaptic loss in the hippocampus. Sevoflurane, an inhalation anesthetic, is widely used in surgical patients and has been associated with neuroinflammation and cognitive impairment, especially in the elderly.^31^ In the perioperative setting, the effects of repeated intravenous administrations of lidocaine on cognitive function following general anesthesia with sevoflurane were investigated. Our MWM test results further indicated that repeated exposure to lidocaine somewhat worsened the working memory impairment induced by sevoflurane in aged mice. Our findings are supported by the results of several human studies that have documented the occurrence of adverse neurological events associated with the administration of intravenous lidocaine.^12^ Andjelkovic et al.^39^ reported delirium in two patients, Dewinter et al.^40^ observed one case of tinnitus and one patient with a metallic taste, and Staikou et al.^41^ documented transient confusion in another patient after anesthesia recovery. Notably, none of the studies recorded plasma lidocaine concentrations exceeding toxic levels.

The age-related response to lidocaine exposure is noteworthy. While aged mice exhibited cognitive impairment after repeated exposure to lidocaine, adult mice did not display similar deficits. Further RNA-seq analysis conducted on the hippocampus revealed a notable number of DEGs that were enriched in inflammatory responses and synaptic signaling after lidocaine exposure in aged mice but not in adult mice. These results suggest that aging may increase vulnerability to lidocaine-induced neurotoxicity, potentially due to age-related alterations in neuronal function and heightened susceptibility to inflammation-induced neurodegeneration.^42^ Additionally, alterations in electrophysiology associated with age intensify the sensitivity of the nervous system to the systemic effects of lidocaine.^43^ Although peak plasma concentrations and protein binding in elderly patients remain largely unchanged, the clearance of local anesthetics is reduced partly because of decreased organ perfusion and metabolic function.^44^ The susceptibility of elderly individuals to lidocaine-induced neurotoxicity emphasizes the significance of age as a crucial determinant when assessing the safety and efficacy of repetitive lidocaine administration, especially in the elderly population. Elucidating the mechanisms underlying this age-dependent response could yield insights into the varying effects of lidocaine on cognitive function among different age groups.

The involvement of astrocyte polarization in mediating lidocaine-induced cognitive impairment is a key finding of this study. Repeated exposure to lidocaine resulted in the transformation of astrocytes from their neuroprotective A2 phenotype to a neurotoxic A1 phenotype in the hippocampus of aged mice. Conversely, a single exposure to lidocaine does not elicit the same effect. BDNF, a crucial neurotrophic factor predominantly secreted by A2 astrocytes, is essential for neuronal survival and growth, synaptic plasticity, and memory formation.^45^ In contrast, reactive A1 astrocytes have been demonstrated to release inflammatory mediators, including C3, leading to impaired neuronal viability and synaptic function. These findings underscore the importance of regulating astrocyte polarization and their interactions with neurons in mediating the neurotoxic effects of lidocaine.

Anesthetics have the ability to modulate microglial activation, leading to both anti- and proinflammatory effects, indicating their potential dual roles in the development of POCD.^46^ Despite recent evidence supporting the anti-inflammatory and neuroprotective effects of lidocaine,^47^^,^^48^ clinical studies have been unable to confirm its effectiveness in reducing the occurrence of POCD. Conversely, lidocaine has been suggested to induce neuroinflammation and is associated with the activation of other immune cells in the rat dorsal root ganglion.^22^ The variability among studies can be attributed to factors including variations in dose, timing, frequency, route of administration of lidocaine, and the type of tissue being studied. Moreover, the inflammatory environment may be influenced by the impact of brain background characteristics. In our study, we observed that repeated exposure to lidocaine induced microglial activation and increased the expression of proinflammatory cytokines in the hippocampus of aged mice. To further evaluate the influence of microglia on astrocytic response and synaptic function following lidocaine exposure, we depleted microglia in aged mice using PLX5622. Our study revealed that pharmacological depletion of microglia effectively prevented the neurotoxic activation of A1 astrocytes and alleviated synaptic impairment. *Consistent with our findings*, previous research has indicated that inhibiting microglia activation^49^ or eliminating microglia^30^ significantly improves synaptic function, thereby underscoring the critical role of microglial activation and neuroinflammation in the development of POCD. Considering the complexity of the pro-inflammatory effects of lidocaine, further research is necessary.

The NF-κB signaling pathway, which is responsible for gene transcription, modulates the expression of inflammatory cytokines and plays a pivotal role in the regulation of inflammation and the immune response.^50^ Accumulating studies have demonstrated the involvement of the NF-κB pathway in mediating microglial activation and synaptic and cognitive impairment in AD models.^51^^,^^52^ However, there is currently no research examining the activation of NF-κB in relation to cognitive dysfunction associated with repeated exposure to lidocaine. In our study, we discovered that the NF-κB pathway and microglia were activated in the hippocampus of aged mice repeatedly exposed to lidocaine. Moreover, posttreatment with DHMEQ effectively inhibited the activation of the microglia and promoted the transition of astrocytes from the A1 to A2 phenotype. Neuronal apoptosis and synapse loss are reported to occur in the hippocampus after anesthesia and surgery,^49^^,^^53^ aligning with our pathological observation. Furthermore, we found that inhibiting NF-κB activation attenuated synaptic loss and neuronal apoptosis and improved cognitive impairment in aged mice following lidocaine exposure. Overall, our findings establish the involvement of the NF-κB pathway in the development of cognitive dysfunction in aged mice subjected to repeated lidocaine exposure and that treatment with an NF-κB inhibitor results in substantial improvements in cognition and mitigates hippocampal pathology.

In conclusion, the findings of this study provide compelling evidence that both age and exposure frequency are positively correlated with the development of cognitive impairment induced by systemic lidocaine, even when administered at a clinically relevant concentration. Moreover, the molecular mechanism underlying synaptic and cognitive impairment in aged mice involves the activation of microglia and neurotoxic A1 astrocytes through the NF-κB signaling pathway. In light of the necessity for certain elderly individuals to undergo multiple surgeries as a preventive measure against recurring illnesses, it is essential to conduct a comprehensive evaluation of the potential benefits and risks linked to repeated intravenous lidocaine.

### Limitations of the study

Several limitations of this study should be acknowledged. First, the study primarily focused on cognitive impairments assessed by the MWM test. However, it is important to explore other cognitive domains, including attention, executive function, and anxiety behavior, to gain a comprehensive understanding of the effects of lidocaine on cognitive function. Second, the study focused on the hippocampus, and other brain regions, such as the prefrontal cortex or the amygdala, could also be affected by lidocaine exposure and should be considered in future investigations. Furthermore, it is crucial to explore various dosing intervals and investigate the long-term effects of lidocaine exposure to advance our understanding and clinical management of cognitive impairments induced by lidocaine.

## Resource availability

### Lead contact

Further information and requests for resources and reagents should be directed to and will be fulfilled by the Lead Contact, Xiaochun Zheng (zhengxiaochun@fjsl.com.cn).

### Materials availability

This study did not generate new unique reagents.

### Data and code availability

• The raw RNA-seq data have been deposited at NCBI and are publicly available. Accession number is listed in the key resources table. The data presented in this paper will be shared by the lead contact upon request.
• This paper does not include the original code.
• Any further information needed to reanalyze the data can be obtained from the lead contact upon request.

## Acknowledgments

This study was supported by funding from the 10.13039/501100001809National Natural Science Foundation of China (No. 82171186), Fujian Research and Training Grants for Young and Middle-aged Leaders in Healthcare, the 10.13039/501100003392Natural Science Foundation of Fujian Province, China (No. 2024J010035), and the Joint Funds for the Innovation of Science and Technology of Fujian Province, China (No. 2023Y9282 and No. 2023Y9315).

## Author contributions

Study supervision: X.Z. and X.C. Study design/planning: X.Z., X.C., H.W., and Y.H. Study implementation: H.W., Y.H., A.C., and X.W. Data analysis: Y.G., J.W., and J.J. Data interpretation: X.C. and P.C. Drafting of manuscript: X.C. and X.Z. Review, editing, and revising: All authors.

## Declaration of interests

The authors declare no competing interests.

## STAR★Methods

### Key resources table

**Table:** 

REAGENT or RESOURCE	SOURCE	IDENTIFIER
**Antibodies**		

Rabbit monoclonal anti-BDNF	Abcam	Cat #ab108319, RRID: AB_10862052
Rabbit monoclonal anti-C3	Abcam	Cat #ab200999, RRID: AB_2924273
Rabbit monoclonal anti-TNF-α	Abcam	Cat #ab183218, RRID: AB_2889388
Rabbit monoclonal anti-C1qA	Abcam	Cat #ab189922, RRID:AB_2894866
Rabbit polyclonal to anti-IL-1α	Abcam	Cat #ab7632, RRID:AB_306001
Rabbit monoclonal anti-S100A10	Abcam	Cat #ab76472, RRID: AB_1524359
Rabbit monoclonal anti-phospho-IκBα	Cell Signaling Technology	Cat #2859, RRID: AB_561111
Rabbit monoclonal anti-IκBα	Cell Signaling Technology	Cat #4812, RRID: AB_10694416
Rabbit monoclonal anti-phospho-NF-κB p65	Cell Signaling Technology	Cat #3033, RRID: AB_331284
Rabbit monoclonal anti-NF-kB p65	Cell Signaling Technology	Cat #8242, RRID: AB_10859369
Rabbit polyclonal anti-Bax	Cell Signaling Technology	Cat #2772, RRID: AB_10695870
Rabbit monoclonal anti-Bcl-2	Cell Signaling Technology	Cat #3498, RRID: AB_1903907
Rabbit polyclonal anti-Cleaved caspase-3	Cell Signaling Technology	Cat #9661, RRID: AB_2341188
Rabbit monoclonal anti-PSD95	Cell Signaling Technology	Cat #3409, RRID: AB_1264242
Rabbit monoclonal anti-synaptophysin	Cell Signaling Technology	Cat #36406, RRID: AB_2799098
Rabbit monoclonal anti-β-actin	Cell Signaling Technology	Cat #8480, RRID: AB_11127855
Mouse monoclonal anti-GFAP	Cell Signaling Technology	Cat #3670, RRID: AB_561049
Rabbit polyclonal anti-Iba-1	Wako	Cat #019-19741, RRID: AB_839504

**Chemicals, peptides, and recombinant proteins**		

Lidocaine	Sigma‒Aldrich	Cat #1366013
CSF1R inhibitor -PLX5622	MedChemExpress	Cat #HY-114153C
NF-κB inhibitor-DHMEQ	ChemScene	Cat #287194-40-5

**Critical commercial assays**		

TRIzol reagent kit	Invitrogen	Cat #10296028CN
QiaQuick PCR extraction kit	Qiagen	Cat #28106
Enhanced chemiluminescence substrate kits	Abcam	Cat #ab133406

**Experimental models: Organisms/strains**		

Mouse: C57BL/6 mice	Shanghai SLAC laboratory Animal Co., Ltd	C57BL/6 colony

**Oligonucleotides**		

RT-qPCR primers, see Table S1	This paper	N/A

**Deposited data**		

Raw bulk RNA-seq data	This paper	GSE270941
SPSS 22.0	IBM SPSS	N/A
imageJ	Olympus	BX50-FLA
Prism 8.0	GraphPad	N/A

### Experimental model and study participant details

#### Animals and ethics statement

The Animal Care and Use Committee at Fujian Medical University granted approval for this research (No: FJMU IACUC 2021-0431, Fuzhou, China). All lab animals were treated in compliance with the Guide for the Care and Use of Laboratory Animals. Experiments were conducted using either young adult (4 months) or aged (18 months) C57BL/6 mice based on their availability. To minimize sex-related bias, each group consisted of an equal distribution of male and female mice. The C57BL/6 mice were housed in plastic cages under controlled ambient and lighting conditions (12-hour light/dark cycle), with unrestricted access to food and water.

### Method details

#### Lidocaine administration and experimental protocol

The present study aimed to determine the applicable dosage and administration route of lidocaine in mice, accounting for established clinical practices and standards. To achieve this goal, lidocaine (Sigma‒Aldrich, St. Louis, MO, USA) was administered intravenously through a tail vein in two groups: the single-dose group (referred to as “Sin lido”) and the repeated-dose group (referred to as “Rep lido”). In the single-dose group, a bolus dose of 18.45 mg kg^-1^ was given, followed by a continuous infusion of 12.30 mg kg^-1^ h^-1^ for 2 hours. In the repeated-dose group, the same regimen was repeated once daily for three consecutive days. According to the body surface area calculation method between mice and humans, these dosages were approximately equivalent to 1.5 mg kg^-1^ and 1 mg kg^-1^ h^-1^ in humans, which are often used in clinical practices.^36^ The control group of mice (referred to as the “Con group”) was administered an equal volume of saline at the corresponding time points.

#### Experiment 1

Initially, we examined the impact of lidocaine exposure on short-term neurocognitive function, taking into account exposure frequency and age by comparing single versus repeated exposures in two age groups: young adults (4 months) and aged mice (18 months). We utilized transcriptome sequencing and bioinformatics analysis to explore the morphopathological alterations occurring in the hippocampus. We assessed the expression of NF-κB pathway-related proteins and examined the responses of microglial and astrocytic cells. Additionally, we evaluated synaptic plasticity, neuronal apoptosis, and learning and memory abilities in mice one day after lidocaine exposure. Furthermore, the effects of repeated intravenous administrations of lidocaine on cognitive function in aged mice following general anesthesia with sevoflurane were investigated. In the "Rep sevo" group, mice were exposed to 2.5% sevoflurane for 2 hours each day over three consecutive days in a repeated exposure protocol. Mice in the "Rep sevo+lido" group received the same sevoflurane exposure regimen along with intravenous lidocaine administrations over the three consecutive days.

#### Experiment 2

To assess the impact of microglia on astrocytic response and synaptic function after lidocaine exposure, we depleted microglia in mice using the CSF1R inhibitor PLX5622, as previously demonstrated.^54^ Achieving a depletion efficiency of over 95%, we started administering PLX5622 (1200 mg/kg, MedChemExpress, NJ, USA) enriched chow to the mice two weeks before the required time point for microglia elimination. The mice were divided into groups receiving either the PLX5622-supplemented diet or a control AIN-76A diet without PLX5622 for two weeks before and after the lidocaine injection, followed by euthanasia. No noticeable behavioral or health issues were evident during the PLX5622 dietary intervention.

#### Experiment 3

Moreover, to explore the role of the NF-κB pathway in microglial activation and the neurotoxic (A1) polarization of astrocytes triggered by repeated lidocaine exposure and its effects on synaptic and cognitive impairment in aged mice, we administered the specific NF-κB inhibitor DHMEQ through intraperitoneal injection. We assessed the levels of proteins involved in the NF-κB pathway, observed alterations in astrocyte phenotype, and evaluated synaptic function, neuronal apoptosis, and learning and memory abilities. The DHMEQ treatment (ChemScene, Monmouth Junction, USA)^55^ was administered concurrently with intravenous lidocaine exposure at a dosage of 4 mg/kg for a duration of 3 days.

#### Assessment of median convulsive thresholds, plasma concentrations and cardiac toxicity after the administration of lidocaine

In our preliminary experiments, we determined the median convulsive dose (CD_50_) of intravenous lidocaine in aged mice by the up-and-down method. We evaluated the convulsive behavior of the mice using a modification of the Racine scale.^56^ Concentrations of plasma lidocaine were measured using high-performance liquid chromatography (HPLC) analysis, as previously described.^57^ Hemodynamic data, including HR and MAP, were recorded to evaluate cardiac toxicity during the administration of lidocaine. Electrocardiograms (ECGs) were obtained from non-anesthetized restrained mice to record HR. The ECG signal was enhanced and digitized using a Picoscope2203 device (Pico Technology Cambridgeshire, UK). MAP was measured using a tail cuff blood pressure (BP) monitor BP monitor.^58^

#### RNA-sequencing and bioinformatics analyses

At 24 hours after exposure to lidocaine, we collected hippocampal tissue from both young and aged mice in the Con and Rep Lido groups. The RNA-seq library preparation and sequencing protocols were performed according to previously described methods.^59^ The overall distribution of differentially expressed genes (DEGs) was depicted using a Volcano plot. To investigate the functional implications of these DEGs in the Rep lido group compared to the Con group, Kyoto Encyclopedia of Genes and Genomes (KEGG) pathway enrichment analysis and gene set enrichment analysis (GSEA) were conducted. These analyses were executed using the OmicShare tool, a freely accessible online platform for data analysis provided by Gene Denovo (Guangzhou, China, http://www.omicshare.com/tools).

#### Real-time quantitative polymerase chain reaction (RT‒qPCR)

RT‒qPCR analysis was conducted to assess the alterations in the mRNA expression of astrocyte phenotype markers in the hippocampus 24 hours after exposure to lidocaine. Briefly, total RNA was extracted using the TRIzol reagent kit (Invitrogen, Carlsbad, CA, USA). Enriched mRNA was then reverse-transcribed into cDNA. The cDNA fragments were purified and subjected to end-repair using the QiaQuick PCR extraction kit (Qiagen, Limburg, the Netherlands). Subsequently, the fragments were poly(A)-tailed and ligated to Illumina sequencing adapters. Agarose gel electrophoresis was conducted to select the PCR amplification products, which were then subjected to sequencing using the NovaSeq6000 platform from Gene Denovo Biotechnology Co (Guangzhou, Guangdong, China). For the specific primer sequences, refer to Table S1.

#### Western blot analysis

Western blot analysis was conducted according to previously described methods.^60^^,^^61^ Hippocampal tissues were harvested 24 hours after exposure to lidocaine and immediately placed on ice. The tissues were then homogenized using a lysis buffer supplemented with protease and phosphatase inhibitors. Equal amounts of proteins were subsequently separated and transferred onto polyvinylidene fluoride membranes. Immunoblots were incubated overnight with primary antibodies targeting BDNF (#ab108319, Abcam, Cambridge, UK), C3 (#ab200999, Abcam), TNF-α (#ab183218, Abcam), C1qA (#ab189922, Abcam), IL-1α (#ab7632, Abcam), phospho-IκBα (Ser32) (#2859, Cell Signaling Technology, Beverly, MA, USA), phospho-NF-κB p65 (Ser536) (#3033, Cell Signaling Technology), NF-κB p65 (D14E12) (#8242, Cell Signaling Technology), IκBα (44D4) (#4812, Cell Signaling Technology), Bax (#2772, Cell Signaling Technology), Bcl-2 (D17C4) (#3498, Cell Signaling Technology), Cleaved caspase-3 (Asp175) (#9661, Cell Signaling Technology), PSD95 (D74D3) (#3409, Cell Signaling Technology), synaptophysin (D8F6H) (#36406, Cell Signaling Technology), and β-actin (#8480, Cell Signaling Technology). Subsequently, the membranes were incubated with secondary antibodies at room temperature for 2 hours. Protein bands were visualized and captured using enhanced chemiluminescence substrate kits (#ab133406, Abcam) and a GE Amersham Imager 600 (AI600; GE Healthcare, Japan).

#### Immunofluorescence staining

We utilized the A1 phenotype marker C3 and the A2 phenotype marker S100A10 to ascertain the transformation of astrocytes. Additionally, we evaluated microglial activation in the hippocampus after exposure to lidocaine by detecting Iba-1 immunofluorescence. Immunofluorescence analysis was performed as previously described.^60^ Brain tissues were sectioned into 3.0–3.5 μm-thick coronal sections and then embedded in paraffin. Subsequently, the denatured DNA was treated with 2 N HCl for 30 minutes at 37°C and neutralized using a 0.1 M borate buffer solution. After blocking with 5% fetal bovine serum, the sections were incubated overnight with primary antibodies targeting C3 (#ab200999, Abcam), S100A10 (#ab76472, Abcam), BDNF (#ab108319, Abcam), GFAP (GA5) (#3670, Cell Signaling Technology), and Iba-1 (#019-19741, WAKO, Osaka, Japan). The following day, the sections were incubated with corresponding secondary antibodies at room temperature for 2 hours. Positive cells were manually counted by a skilled laboratory technician using a fluorescence microscope (Olympus FV1000, Japan) and the cell counter function of ImageJ 1.4.

#### Transmission electron microscopy

The ultrastructure of neural synapses was observed using a transmission electron microscope (TEM) as previously described.^62^ Hippocampal tissues were harvested 24 hours after exposure to lidocaine. Briefly, the hippocampal tissues were fixed in 2.5% glutaraldehyde for 2 hours and subsequently postfixed in 1% osmium tetroxide for another 2 hours. The sample was then dehydrated using ethyl alcohol gradients and subjected to embedding, sectioning, and staining with uranyl acetate and lead citrate. Finally, observation was conducted using a transmission electron microscope (JEM-2100, Jeol, Japan) at ×40,000 magnification. The thickness of postsynaptic density (PSD) were analyzed using imageJ software (BX50-FLA, Olympus, Japan).

#### Morris water maze test

Hippocampus-dependent spatial learning and memory abilities were evaluated using the MWM test 24 hours after lidocaine exposure, as described previously.^62^ The MWM test consisted of a place navigation test conducted over the first five days, followed by a probe trial on the sixth day. To minimize diurnal variations and the influence of light on the experimental outcomes, all tests were performed between 9:00 am. and 3:00 pm. Mice movements were recorded using a video camera positioned above the pool, which was divided into four quadrants with one quadrant containing a submerged platform. During the place navigation test, mice were positioned facing the pool wall and given 60 seconds to search for the escape platform. We repeated this experiment four times per day for five days and measured the average escape latency as an indicator of spatial learning ability. In the probe trial, we removed the hidden platform and allowed the mice to swim freely in the pool for 60 seconds. As part of our assessment of their spatial memory ability, we recorded the number of platform crosses and the time spent in the target quadrant.

### Quantification and statistical analysis

The data were analyzed using SPSS version 22.0 software (SPSS Inc., Chicago, USA). The normality of the data distribution was assessed using the Shapiro‒Wilk test, while Levene's test was utilized to evaluate the homogeneity of variance. When the data exhibited a normal distribution, they were presented as the mean ± standard deviation (SD). Multiple group comparisons were conducted using one-way analysis of variance (ANOVA), and a Bonferroni correction was applied if the data followed a normal distribution. However, if the data did not conform to a normal distribution, a nonparametric Kruskal‒Wallis test was employed. The escape latency in the MWM test was evaluated using repeated measures two-way ANOVA with “day” as the within-subject factor and “group” as the between-subject factor. *p* < 0.05 was considered statistically significant.
