# Supplementary material for: Repeated lidocaine exposure induces synaptic and cognitive impairment in aged mice by activating microglia and neurotoxic A1 astrocytes
Source: iScience. 2025 Feb 17;28(3):112041. doi: 10.1016/j.isci.2025.112041 (PMC11910116; doi:10.1016/j.isci.2025.112041)
Supplement: Document S1. Figures S1–S3 and Table S1 [file mmc1.pdf]

iScience, Volume *28*

Supplemental information

Repeated lidocaine exposure induces synaptic and cognitive impairment in aged mice by activating microglia and neurotoxic A1 astrocytes

Xiaohui Chen, Haiyang Wan, Yongxin Huang, Andi Chen, Xuyang Wu, Yanhua Guo, Jianjie Wei, Pinzhong Chen, Jiangdan Jiang, and Xiaochun Zheng

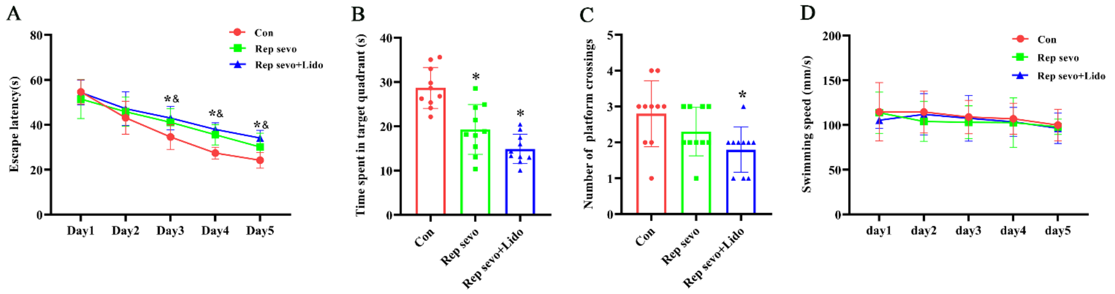

Figure S1. Repeated exposure to lidocaine worsened the working memory impairment induced by sevoflurane in aged mice, related to Figure 2.

(A) Escape latency of aged mice to find the hidden platform during the 5-day training trials in the MWM test.

(B) Time spent in the target quadrant during the probe test.

(C) Number of platform crossings of aged mice during the probe test.

(D) Swim velocity of aged mice during the 5-day training trials.

Con: control group; Rep sevo: repeated sevoflurane exposure group. Rep sevo + Lido: group that received repeated exposure to both sevoflurane and lidocaine. n = 10 per group. *$p < 0.05$ compared with the con group, $^{\#}p < 0.05$ compared with the Rep sevo group. Data were presented as the mean ± SD.

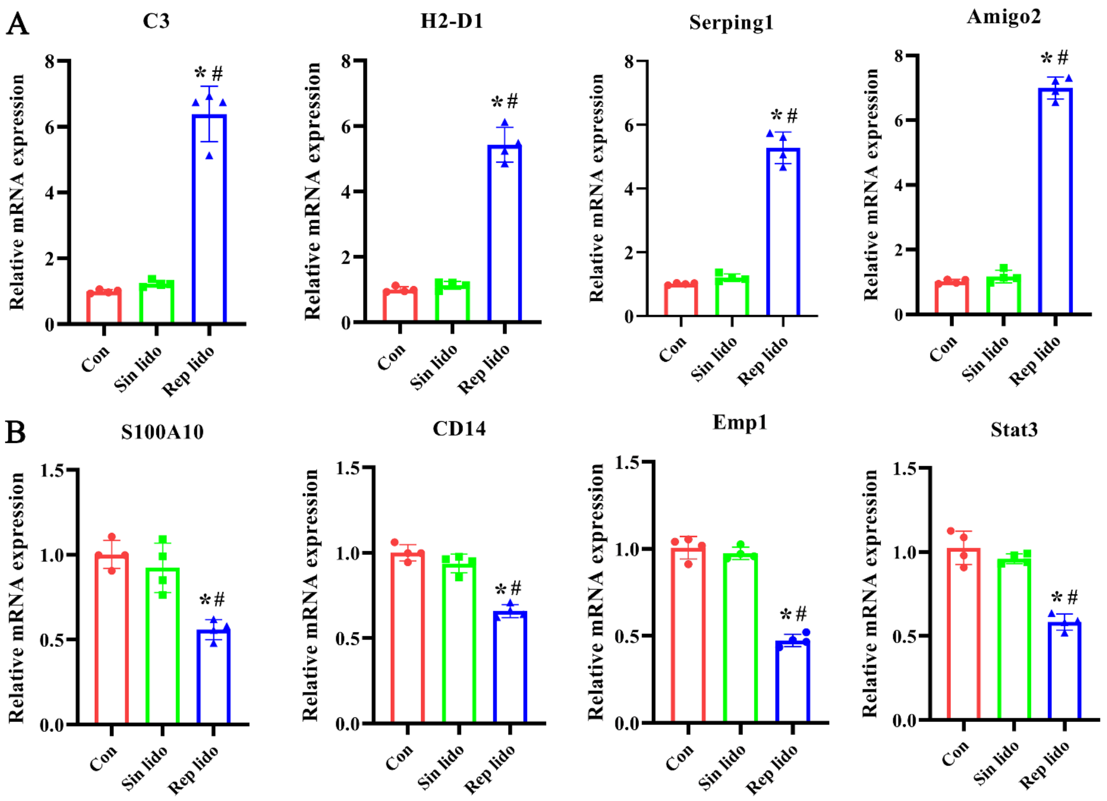

Figure S2. Repeated lidocaine exposure promotes the transformation of astrocytes from the A2 phenotype to the A1 phenotype in the hippocampus of aged mice, related to Figure 5.

(A) RT–qPCR analyses showed a significant increase in the levels of astrocytes A1-specific transcripts (C3, H2-D1, Serping1, Amigo2) and (B) A decrease in the levels of A2-specific genes (S100A10, CD14, Emp1, Stat3) in the hippocampus of aged mice after repeated lidocaine exposure.

Con: control group; Sin lido: single lidocaine exposure; Rep lido: repeated lidocaine exposure. n = 4 per group. *$p < 0.05$ compared with the con group, $^{\#}p < 0.05$ compared with the sin lido group. Data were presented as the mean ± SD.

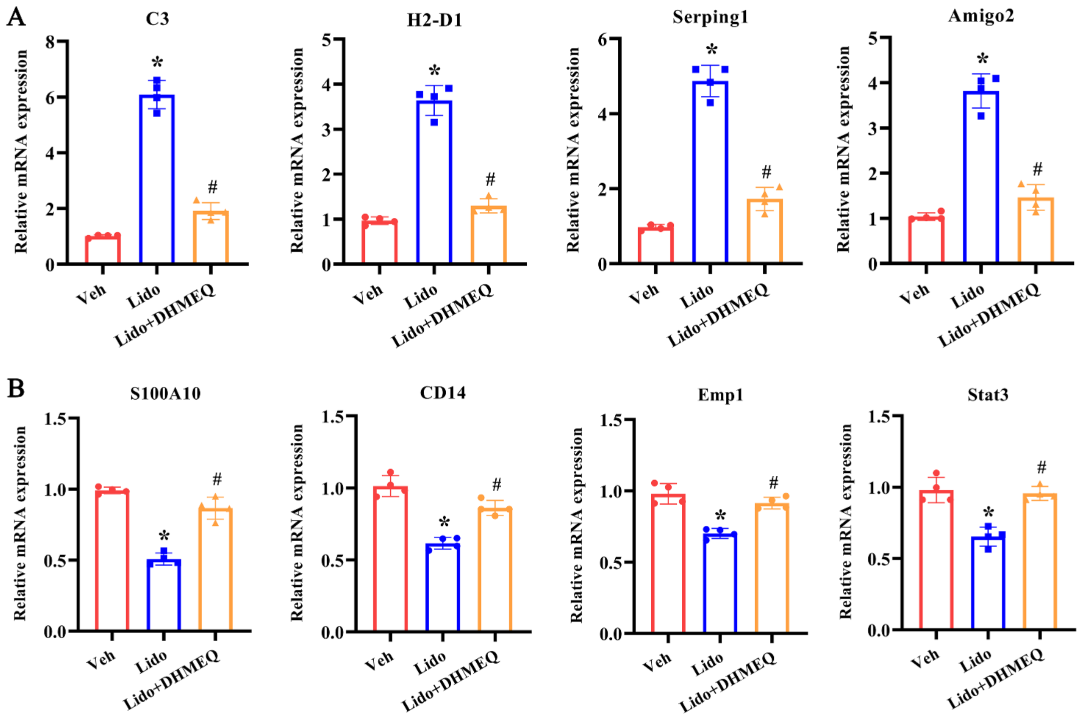

Figure S3. DHMEQ treatment prevented A1 astrocyte polarization induced by repeated lidocaine exposure in the hippocampus of aged mice, related to Figure 8.

(A) mRNA expression of astrocytes A1-specific genes (C3, H2-D1, Serping1 and Amigo2) and (B) neuroprotective markers (S100A10, CD14, Emp1 and Stat3) in the hippocampus detected by RT–qPCR analysis.

Veh: vehicle group; lido: group that received repeated lidocaine exposure. n=4 per group. *$p < 0.05$ compared with the veh group, #$p < 0.05$ compared with lido group. Data were presented as the mean ± SD.

Table S1. The forward and reverse primers for RT-qPCR

**Genes**	Primers	**Sequence (5′–3′)**
GAPDH	Forward primer	TGGAAAGCTGTGGCGTGATG
	Reverse primer	TACTTGGCAGGTTTCTCCAGG
C3	Forward primer	AGAATCGCTACTTCCAGACCAT
	Reverse primer	CTTGACCTCCACCTCTTGTTG
Serping1	Forward primer	TGCCCACTTACCTGACGATG
	Reverse primer	AGTTCCAGCACTGTCTCGTG
Amigo2	Forward primer	CGTAGGCACTTTAGCTCCGT
	Reverse primer	CGTGGATAAAGCCAAGTGCG
H2-D1	Forward primer	GCTCTCACACACTCCAGCAGAT
	Reverse primer	CTCTGCTCCCACTTGCGT
S100a10	Forward primer	AGTGCTCATGGAACGGGAGT
	Reverse primer	CTTTGCCATCTCGGCACTGG
Emp1	Forward primer	CCTTGTGGTCTTCGTGTTCC
	Reverse primer	GGCGTAATGATGAGTGTAGATTGA
Cd14	Forward primer	CAAGTTCCCGACCCTCCAAG
	Reverse primer	GCATCCCGCAGTGAATTGTG
Stat3	Forward primer	AGCTGGACACACGCTACCT
	Reverse primer	AGGAATCGGCTATATTGCTGGT
TNF-α	Forward primer	GCCGATGGGTTGTACCTTGT
	Reverse primer	TCTTGACGGCAGAGAGGAGG
